# Pulp Stones

**Published:** 1889-05

**Authors:** J. M. Clyde

**Affiliations:** Springfield, Mo.


### "Pulp Stones."

BY J. M. CLYDE, D.D.S., SPRINGFIELD, MO.

Recently a gentleman about fifty years of age called on me complaining of severe neuralgia. He had no idea that it was caused by the tooth about to be described. The tooth was the second lower molar, left side. It had a medium sized cavity on the anterior proximal surface and was exceedingly sensitive to cold water. After removing the carious material, I filled with cement and awaited further development. This treatment did not improve matters in the least. In the meantime I filled several other teeth, removed tartar, etc., but still the neuralgia continued as before. Suspicion pointing to the aforesaid molar sufficiently to warrant the operation, I exposed the pulp and applied arsenic, which soon ended the trouble. On removing the pulp I discovered two granules at the anterior buccal horn of the pulp and the chamber considerably enlarged at this point. I presume the enlargement was the result of retrogressive metamorphosis of the dentine. I met a case similar to this several years ago.

If you think there is any thing in this which may be of practical value to others, please give it place in your columns.

